# Social Regulation of Gene Expression in Threespine Sticklebacks

**DOI:** 10.1371/journal.pone.0137726

**Published:** 2015-09-14

**Authors:** Anna K. Greenwood, Catherine L. Peichel

**Affiliations:** Divisions of Basic Sciences and Human Biology, Fred Hutchinson Cancer Research Center, Seattle, Washington, United States of America; University of Calgary, CANADA

## Abstract

Identifying genes that are differentially expressed in response to social interactions is informative for understanding the molecular basis of social behavior. To address this question, we described changes in gene expression as a result of differences in the extent of social interactions. We housed threespine stickleback (*Gasterosteus aculeatus*) females in either group conditions or individually for one week, then measured levels of gene expression in three brain regions using RNA-sequencing. We found that numerous genes in the hindbrain/cerebellum had altered expression in response to group or individual housing. However, relatively few genes were differentially expressed in either the diencephalon or telencephalon. The list of genes upregulated in fish from social groups included many genes related to neural development and cell adhesion as well as genes with functions in sensory signaling, stress, and social and reproductive behavior. The list of genes expressed at higher levels in individually-housed fish included several genes previously identified as regulated by social interactions in other animals. The identified genes are interesting targets for future research on the molecular mechanisms of normal social interactions.

## Introduction

Social interactions with conspecifics are found across all animal taxa, and the fundamental processes that govern social behavior are highly conserved. Among vertebrates, the core brain circuitry and key neuropeptides and neuromodulators that mediate social behavior are shared ([1,2]; but see [3]). Furthermore, recent work has shown that gene networks that regulate social behavior are even conserved across invertebrates and mammals [4].

To identify genes and molecular pathways involved in social behavior, previous studies have examined animals with different social experiences to determine which genes show changes in expression [5–7]. These studies have either examined the expression of candidate genes or have employed expression arrays or transcriptome sequencing to more globally sample gene expression changes [5–8]. Global expression studies in vertebrates have identified numerous genes that are socially regulated, highlighting genes not previously associated with social behavior [4,8–15]. These studies have been informative for dissecting the molecular mechanisms of sociality [4,5].

Here we sought to identify the genes that play a role in normal interactions among fish in a social group. We used threespine sticklebacks (*Gasterosteus aculeatus*), which are a longstanding model for studies of social behavior and have a wealth of genomic resources available, which facilitates transcriptomic analyses [16,17]. Marine sticklebacks are highly social, and are typically found in social groups [17,18]. We modulated the extent of social interactions of individual fish by housing fish either in social groups or individually for a one-week period. This manipulation should permit detection of a state change that is not due to the process of isolation (i.e. not within several hours), but also avoids the detrimental effects of long-term isolation on increasing stress and anxiety [19]. We then used RNA-sequencing (RNA-seq) to compare gene expression in brains of group- or individually-housed fish.

## Materials and Methods

### Fish and sample collection

Fish were from a lab-reared population of Japanese Pacific Ocean marine fish originally derived from the Bekanbeushi River in Japan. Fish were reared in 110-L tanks in 3.5 ppt seawater (Instant Ocean, United Pet Group, Blacksburg, VA) at 16 C, and under 16 h light / 8 h dark lighting conditions. Fish were fed *Artemia* nauplii and mysis shrimp. All fish were treated in accordance with the guidelines of the Institutional Animal Care and Use Committee of the Fred Hutchinson Cancer Research Center (FHCRC), protocol number 1575.

For social housing manipulation, fish from a single community tank were caught and transferred to four new 38-L tanks. Fish were either housed individually (n = 2 tanks) or in groups of eight mixed sex fish (n = 2 tanks). After one week of individual or group housing, we removed a single fish from each tank for analysis such that we had two individually-housed and two group-housed fish. We replicated this experiment with a second tank of fish so that we had a total of four biological replicates for both individually- and group-housed fish, from two original home tanks. Gonads were visually inspected to identify sex and maturity. Only pre-reproductive females were included in the experiment. Fish were euthanized with MS-222 and their brains were removed into RNA-later (Life Technologies, Carlsbad, CA) and stored at -20 C. Brains of individual fish were then dissected into three portions: 1) the telencephalon, 2) the diencephalon, pituitary, and rostral midbrain, and 3) the caudal midbrain, hindbrain, and cerebellum. We will refer to these portions as telencephalon, diencephalon, and hindbrain/cerebellum for simplicity. Tissue was homogenized using a pellet pestle (Kimble-Chase, Vineland, NJ) and total RNA was isolated using Trizol (Life Technologies, Carlsbad, CA). We performed the dissection and RNA isolation in separate batches on two different days, such that fish from one experimental replicate (i.e. home tank of origin) were processed on the same day.

### RNA-seq

Barcoded RNA libraries from 24 samples (eight fish each with three brain regions) were generated in the FHCRC Genomics facility using Illumina’s TruSeq RNA Sample Prep Kit v2 (Illumina, San Diego, CA) and a Sciclone NGS Workstation (PerkinElmer, Waltham, MA). Libraries were multiplexed, split across three lanes, and 50-bp paired-end sequences were generated on an Illumina HiSeq 2500 (Illumina, San Diego, CA). Demultiplexing was performed using Illumina's CASAVA v1.8.2 software, allowing for a single mismatch in the index read. Fastq files have been deposited to the Sequence Read Archive (Study Accession SRP056943). We used a local instance of Galaxy [20–22] to perform alignment and to quantify reads aligning to genes. Reads were first aligned to the stickleback genome (BroadS1 [16]) using the default parameters in tophat2 (version 2.0.9, Galaxy tool version 0.6 [23]). Next, reads that fell within predicted genes (Ensembl genes 76) were counted using htseq-count (“Count reads in features with htseq-count” Galaxy tool v1.0 [24]). In htseq-count, we used the following parameters:-q-m intersection-nonempty-s no-a 0-t exon-i gene_id. The resulting matrix was exported from Galaxy and imported into R (http://r-project.org) where we used edgeR, version 3.8.6, [25] to identify differentially expressed genes. A multidimensional scaling (MDS) plot was generated in edgeR. We also calculated the biological coefficient of variation (BCV) of samples using edgeR.

We first analyzed expression differences as a function of brain region, independent of social environment, by performing three analyses: telencephalon *vs*. diencephalon and hindbrain/cerebellum; diencephalon *vs*. telencephalon and hindbrain/cerebellum; and hindbrain/cerebellum *vs*. telencephalon and diencephalon. We filtered out genes that did not have at least 1 count per million reads in at least two samples. We present and discuss the top 10 differentially expressed genes for each brain region, all of which were significant at a False Discovery Rate (FDR) of *P* < 0.05.

To identify genes differentially expressed as a function of social environment, we next performed a General Linear Model (GLM) analysis separately for each brain region by comparing read counts in group- and individually-housed fish. We included experimental replicate (1 or 2) as a factor in the model to control for home tank of origin and RNA isolation-batch effects. We filtered out genes that did not have at least 1 count per million reads in at least two samples. Differentially expressed genes were those that had FDR of 0.05. We present and discuss the genes upregulated in group- and individually-housed fish separately, so for simplicity we report the log 2 fold change (log2FC) as positive for both comparisons.

### Functional annotation and enrichment analysis

We used DAVID to perform functional annotation and enrichment analysis [26]. DAVID tests enrichment of Gene Ontology (GO) terms, as well as other annotation categories including Interpro domains, KEGG pathways, and SMART protein domains. Ensembl gene identifiers were first converted to zfin identifiers specifically for these analyses. Fold-enrichment of all significant up- or down-regulated genes was calculated over the background gene list, which included all genes expressed in the hindbrain/cerebellum. Functional annotation terms that were significantly enriched are reported, and are organized into clusters based on DAVID’s functional annotation clustering.

We also tested for enrichment of glutamate receptors in genes upregulated in group-housed fish. We counted the number of glutamate receptor and GABA receptor genes in the upregulated list and the list of all genes expressed in the hindbrain/cerebellum. We then used the test of equal proportions in R to determine whether there was significant enrichment of these gene classes.

## Results and Discussion

Sequencing generated an average of 42 ± 2 million total reads per sample, of which 88 ± 1% aligned to the genome. Of the aligned reads, 40 ± 2% fell within a predicted gene, thus were counted by htseq-count, and included in the analysis. Genes expressed at low levels were not included, leaving a total of 17,095 genes for the telencephalon, 17,553 for the diencephalon, and 17,081 for the hindbrain/cerebellum.

### Differential expression as a function of brain region

**Fig 1:**
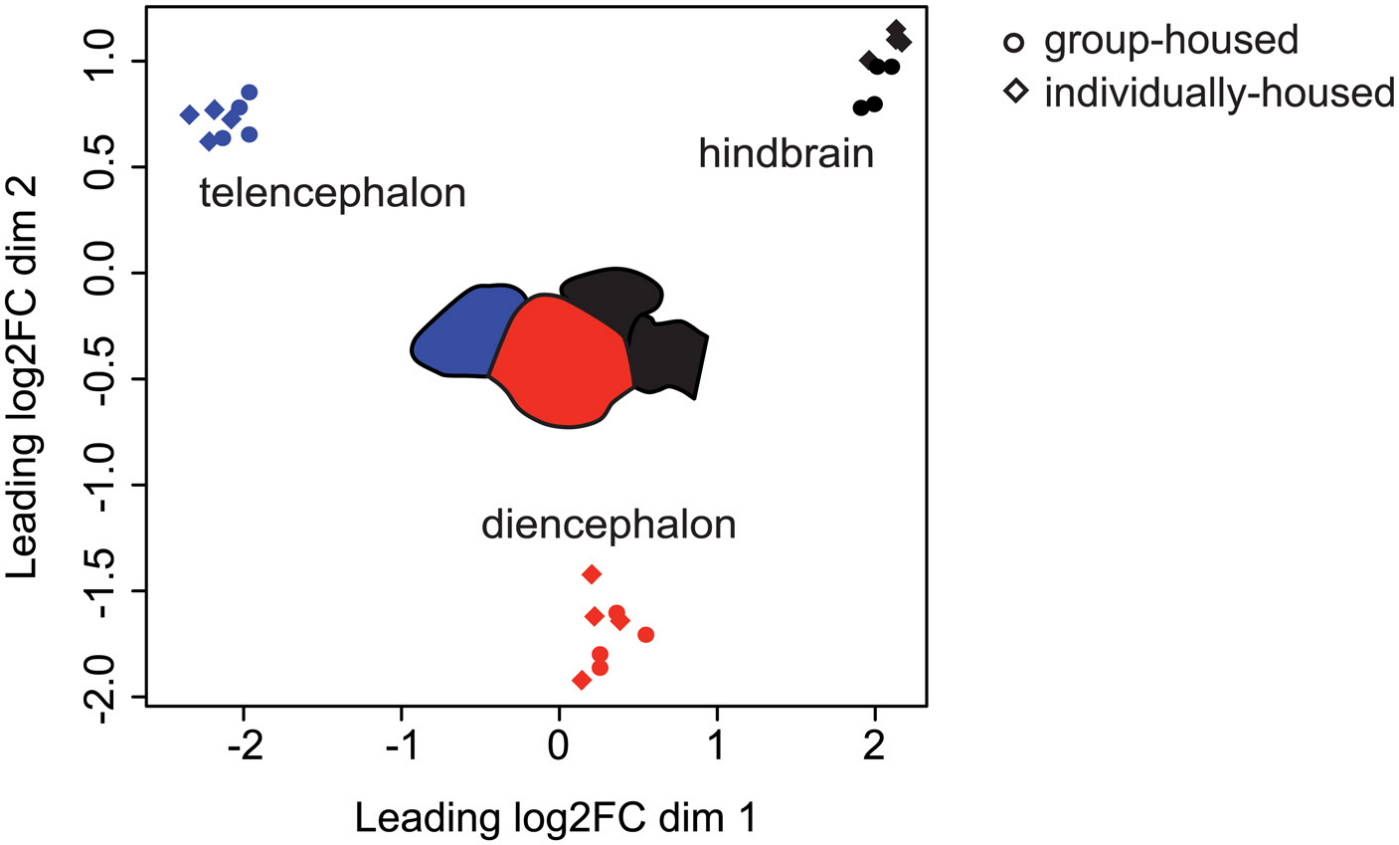
Multidimensional scaling plot reveals separation of samples based on brain region. Multidimensional scaling plot shows leading log2 fold-change (log2FC) differences between samples. Brain regions are colored as follows: blue = telencephalon; red = diencephalon; black = hindbrain/cerebellum. Inset shows schematic of brain with the same colors representing dissected brain regions. Circles = group-housed samples; diamonds = individually-housed samples.

We first compared gene expression as a function of brain region, independent of social housing condition. A multidimensional scaling plot showed clear separation of samples based on brain region (Fig 1). The top ten differentially expressed genes in each brain area based on log2FC included genes with known functions in these parts of the brain (Table 1). For example, the top ten genes enriched in the hindbrain/cerebellum were all known or predicted homeobox (*Hox*) transcription factors (Table 1). *Hox* genes are involved in hindbrain patterning during development and are expressed in the adult brain [27]. Eight of the top ten differentially expressed genes in the diencephalon encode pituitary hormones (Table 1), which was expected as this portion of the brain contained the pituitary. The other two diencephalon-enriched genes were *Nr5a1a*, which is expressed in the diencephalon of zebrafish [28] and *Mibp*, whose function in the brain has not been studied. In the telencephalon, the top ten differentially expressed genes included: 1) genes that are known to be involved in forebrain patterning and/or used as forebrain markers (*Eomesa* and *Emx3* [29], *Tbr1b* [30], *Scgn* [31]), 2) a gene expressed in the forebrain of zebrafish (*Rtn4rl2b* [32]), and 3) genes with unclear function in the brain (*Apod*, *Ctrb1*). The genes identified as being highly enriched in specific brain regions may prove to be useful markers of different neuronal populations in future neuroanatomy studies in sticklebacks and other fish.

**Table 1:** Top ten genes enriched in each brain region.

Ensembl Gene ID	Log2FC	FDR	Symbol	Description
**Telencephalon**				
ENSGACG00000017663	8.7	1.57E-110	Ctrb1	Chymotrypsinogen B1
ENSGACG00000016370	8.5	2.37E-108	Emx3	Empty spiracles homeobox 3
ENSGACG00000016991	7.2	8.62E-38	Apod	Apolipoprotein D
ENSGACG00000005648	6.6	2.31E-87	Tbr1b	T-box, brain, 1b
ENSGACG00000003160	6.5	0	Eomesa	Eomesodermin homolog a
ENSGACG00000018955	6.4	1.44E-23	NA	Protein family: Solute Carrier Family 12
ENSGACG00000003159	6.4	2.07E-142	NA	Novel protein
ENSGACG00000009609	6.2	8.14E-73	Scgn	Secretagogin
ENSGACG00000013917	6.0	1.49E-24	NA	Novel protein
ENSGACG00000017836	6.0	5.99E-56	Rtn4rl2b	Reticulon 4 receptor-like 2b
**Diencephalon**				
ENSGACG00000009153	9.0	2.57E-133	Cga	Glycoprotein hormones, alpha polypeptide
ENSGACG00000006561	9.0	5.05E-248	Prl	Prolactin
ENSGACG00000014829	8.9	2.75E-153	Gh1	Growth hormone 1
ENSGACG00000009521	8.7	1.07E-239	Pomca	Proopiomelanocortin a
ENSGACG00000018017	8.4	2.54E-284	Pmchl	Pro-melanin-concentrating hormone, like
ENSGACG00000018317	8.4	1.05E-76	Nr5a1a	Nuclear receptor subfamily 5, grp A, mbr 1b
ENSGACG00000006593	8.0	1.76E-126	Smtla	Somatolactin alpha
ENSGACG00000005276	7.9	6.85E-70	Tshb	Thyroid stimulating hormone, beta subunit
ENSGACG00000011475	7.7	1.80E-18	Lhb	Luteinizing hormone, beta polypeptide
ENSGACG00000015226	7.5	8.56E-37	Mipb	Major intrinsic protein of lens fiber b
**Hindbrain/Cerebellum**				
ENSGACG00000009421	9.7	2.69E-47	Hoxc4a	Homeobox c4a
ENSGACG00000007108	9.0	1.48E-27	Hoxa5a	Homeobox a5a
ENSGACG00000007100	8.1	3.51E-33	Hoxa4	Homeobox a4
ENSGACG00000004548	7.5	3.86E-64	Hoxd3a	Homeobox d3a
ENSGACG00000004551	7.2	1.10E-28	Hoxd4a	Homeobox d4a
ENSGACG00000009416	7.2	3.84E-17	Hoxc5a	Homeobox c5a
ENSGACG00000005631	7.0	3.11E-43	Hoxb3a	Homeobox b3a
ENSGACG00000005626	6.9	1.54E-10	NA	Protein family: Homeobox
ENSGACG00000003945	6.7	2.24E-12	Hoxb5b	Homeobox b5b
ENSGACG00000005633	6.6	6.06E-42	Hoxb2a	Homeobox b2a

Log2FC = log2 fold-change, FDR = false discovery rate, Symbol = gene name, NA = novel gene with no associated name.

### Differential expression as a function of social housing

We next identified genes that were differentially expressed as a result of social experience. There were numerous genes that were differentially expressed in the hindbrain/cerebellum (985 higher in group and 401 higher in isolate; all significant genes are shown in S1 File; the top 25 are shown in Tables 2 and 3). However, few genes were differentially expressed in either the diencephalon (5 higher in group) or telencephalon (1 higher in isolate). Four of the five differentially expressed genes in the diencephalon (Table 2) were also upregulated in the hindbrain/cerebellum of group-housed fish (S1 File; hindbrain/cerebellum values: *Cyr61*: log2FC = 2.4; FDR < 0.008, *Tgm8*: log2FC = 1.5; FDR = 0.008, *Etv5a*: log2FC = 0.9; FDR < 0.001, and *Fam46d*: log2FC = 1; FDR < 0.012). The fifth gene, novel gene ENSGACG00000012907, was not differentially expressed in the hindbrain/cerebellum (log2FC = 0.3; FDR = 0.13). *Etv5a* is a transcription factor involved in specification of dopaminergic cells in *C*. *elegans*, and has been shown to co-localize with diencephalic dopaminergic cell populations in fish [33]. *Cyr61* is expressed at the midbrain-hindbrain boundary in developing zebrafish, but its function is unknown [34]. *Tgm8* shares distant homology with the transglutaminase family, which are enzymes involved in protein cross-linking [35]. *Tgm8* was highly differentially expressed in all three brain regions, although it did not reach an FDR threshold of p < 0.05 in the telencephalon (higher in group; log2FC = 1.9; FDR = 0.13). *Fam46d* has an unknown neural function but is known to be expressed at higher levels in a mouse model of autism [36]. The single gene that was differentially expressed in the telencephalon is *Proca1*, whose function is unknown other than it is found in a protein complex with the cell division gene cyclin A1 (Table 3).

**Table 2:** Genes significantly upregulated in group-housed fish.

Ensembl Gene ID	Log2FC	FDR	Symbol	Description
**Diencephalon**				
ENSGACG00000017235	3.0	0.001	Cyr61	Cysteine-rich, angiogenic inducer, 61
ENSGACG00000003741	1.8	0.000	Tgm8	Transglutaminase 8
ENSGACG00000008646	1.3	0.000	Etv5a	Ets variant 5a
ENSGACG00000018558	0.8	0.041	Fam46d	Family with sequence similarity 46, member D
ENSGACG00000012907	0.8	0.041	NA	Novel protein
**Hindbrain/Cerebellum**				
ENSGACG00000007463	3.9	0.025	Syne2a	Spectrin repeat containing, nuclear envelope 2a
ENSGACG00000005626	3.4	0.001	Hoxb5	Homeobox B5
ENSGACG00000001172	3.2	0.002	NA	Protein family: Histone lysine N methyltransferase
ENSGACG00000005716	3.1	0.017	NA	Protein family: Hyaluronidase
ENSGACG00000018064	3.1	0.008	NA	Novel protein
ENSGACG00000003170	3.0	0.047	NA	Protein family: Multiple PDZ domain
ENSGACG00000002950	3.0	0.044	Szt2	Seizure threshold 2 homolog
ENSGACG00000017590	2.9	0.007	Crema	cAMP responsive element modulator a
ENSGACG00000003945	2.9	0.003	Hoxb5b	Homeo box B5b
ENSGACG00000002005	2.9	0.015	NA	Novel protein
ENSGACG00000013776	2.7	0.005	Herc2	Hect domain and RLD 2
ENSGACG00000001636	2.7	0.044	NA	Novel pseudogene
ENSGACG00000011127	2.7	0.048	Stard9	StAR-related lipid transfer domain containing 9
ENSGACG00000009610	2.6	0.008	NA	Novel protein
ENSGACG00000008919	2.5	0.013	Kcnk9	Potassium channel, subfamily K, member 9
ENSGACG00000018488	2.5	0.004	NA	Protein family: High affinity choline transporter 1
ENSGACG00000007108	2.4	0.002	Hoxa5a	Homeo box A5a
ENSGACG00000009416	2.4	0.020	Hoxc5a	Homeo box C5a
ENSGACG00000014677	2.4	0.009	Prrc2c	Proline-rich coiled-coil 2C
ENSGACG00000011293	2.4	0.015	Hectd4	HECT domain containing E3 ubiquitin ligase 4
ENSGACG00000004479	2.4	0.008	Sst1.1	Somatostatin 1, tandem duplicate 1
ENSGACG00000011057	2.4	0.004	NA	Novel protein
ENSGACG00000004861	2.4	0.012	Agrn	Agrin
ENSGACG00000007999	2.4	0.038	Rarb	Retinoic acid receptor, beta
ENSGACG00000004506	2.3	0.008	S100u	S100 calcium binding protein U

All five significant genes from diencephalon and top 25 from hindbrain/cerebellum are shown; no genes were significantly upregulated in the telencephalon. Log2FC = log2 fold-change, FDR = false discovery rate, Symbol = gene name, NA = novel gene with no associated name.

**Table 3:** Genes significantly upregulated in individually-housed fish.

Ensembl Gene ID	Log2FC	FDR	Symbol	Description
**Telencephalon**				
ENSGACG00000011223	2.3	0.003	Proca1	Protein interacting with cyclin A1
**Hindbrain/Cerebellum**				
ENSGACG00000001322	2.5	0.029	NA	Novel protein
ENSGACG00000005350	2.2	0.037	Slc16a1	Solute carrier family 16 member 1
ENSGACG00000017681	2.0	0.043	Pmt	Phosphoethanolamine methyltransferase
ENSGACG00000004653	2.0	0.045	NA	Novel protein
ENSGACG00000001231	1.9	0.019	NA	Novel protein
ENSGACG00000021449	1.9	0.006	NA	Novel miRNA
ENSGACG00000002911	1.9	0.004	Tcf24	Transcription factor 24
ENSGACG00000001910	1.6	0.027	NA	Protein family: MHC class I antigen
ENSGACG00000007674	1.5	0.047	NA	Protein family: Glutathione S transferase
ENSGACG00000008596	1.4	0.011	Ddit4	DNA-damage-inducible transcript 4
ENSGACG00000004576	1.4	0.028	Mad2l1bp	Mad2l1 binding protein
ENSGACG00000022181	1.3	0.003	NA	Novel miRNA
ENSGACG00000007379	1.3	0.000	Stmn1b	Stathmin 1b
ENSGACG00000015933	1.3	0.025	Clec18b	C-type lectin domain family 18, member B
ENSGACG00000012872	1.2	0.019	Eps8l1	Eps8-like1
ENSGACG00000011011	1.2	0.015	NA	Novel protein
ENSGACG00000018331	1.2	0.009	Mxd3	MAX dimerization protein 3
ENSGACG00000002889	1.2	0.044	Sox1b	SRY-box containing gene 1b
ENSGACG00000021538	1.2	0.040	NA	Novel miRNA
ENSGACG00000017065	1.1	0.048	Clul1	Clusterin-like 1 (retinal)
ENSGACG00000006502	1.1	0.048	Parp6b	Poly (ADP-ribose) polymerase, member 6b
ENSGACG00000019774	1.1	0.020	NA	Novel protein
ENSGACG00000015028	1.1	0.048	Gatm	Glycine amidinotransferase
ENSGACG00000015636	1.1	0.000	Cdk2ap1	Cyclin-dependent kinase 2 associated protein 1
ENSGACG00000015171	1.1	0.002	NA	Novel protein

One significant gene from telencephalon and top 25 from hindbrain/cerebellum are shown. Log2FC = log2 fold-change, FDR = false discovery rate, Symbol = gene name, NA = novel gene with no associated name.

It was interesting that many genes were differentially expressed in the hindbrain/cerebellum compared with few in either the telencephalon and diencephalon, which both contain nuclei known to be involved in the control of social behavior [37]. There are several possible explanations for this result. First, the hindbrain and cerebellum may indeed show a greater response to this alteration in social housing than the rest of the brain. Social interactions are associated with sensory stimulation, and this is reduced in individually-housed fish. The hindbrain serves as a primary sensory relay for several senses, and thus may show an increased transcriptional response to this manipulation. Alternatively, lack of detection of differentially expressed genes in the telencephalon and diencephalon could theoretically result from increased heterogeneity of these regions compared with the hindbrain/cerebellum. However, the coefficient of variation is similar across all brain regions (telencephalon BCV = 0.212; diencephalon BCV = 0.206; hindbrain/cerebellum BCV = 0.201), suggesting that this is not the cause in this case. Moreover, another study of stickleback gene expression differences that dissected the brain into similar portions did detect gene expression differences in all regions 30 min after social stimulation [14]. In that study, the diencephalon had the largest number of differentially expressed genes, whereas the telencephalon had the fewest. Thus, it is likely that there are differences in which brain regions respond to different stimuli. In addition, timing of stimulus exposure likely has an important impact on differential gene expression; this should be tested more thoroughly in future studies.

### Genes upregulated in the hindbrain of group-housed fish

The 25 genes that were higher in the hindbrain of group-housed fish, based on fold-change, are shown in Table 2. Many of these genes were involved in developmental processes. The *Hox* genes and retinoic acid receptor (*Rarb*) are specifically involved in hindbrain development [38]. Several additional genes are otherwise implicated in neural development (*Agrn* [39] and *Syne2a* [40]) or intellectual disability (*Herc2* [41] and *Kcnk9* [42]), and *Stard9* is involved in cell division [43]. Functional annotation and enrichment analysis echoed the finding that developmental genes are strongly enriched in the list of genes upregulated in group-housed fish (Table 4). All of the significantly enriched functional clusters were related to development, including cell morphogenesis and neural development, cell adhesion, plexin/semaphorin signaling, and EGF signaling (Table 4). Semaphorins and EGF signaling are involved in neural development [44,45]. Increased activity of developmental processes is suggestive of more arborization and neurogenesis in group-housed fish. There is ongoing neurogenesis in the hindbrain/cerebellum of sticklebacks [46], and previous work has shown that sensory stimulation, including social housing, can alter levels of neurogenesis in other fish [47]. It is possible that the upregulated gene expression of developmental genes in the hindbrain/cerebellum of group-housed fish is related to increased sensory function due to higher levels of sensory stimulation.

**Table 4:** Functional annotation and clustering of genes expressed at higher levels in group-housed fish.

Cluster	Term	Description	Fold Enrichment
1	GO:0000904	Cell morphogenesis involved in differentiation	4.2
1	GO:0007409	Axonogenesis	4.1
1	GO:0048667	Cell morphogenesis involved in neuron differentiation	4.1
1	GO:0032989	Cellular component morphogenesis	2.9
1	GO:0048812	Neuron projection morphogenesis	4.1
1	GO:0000902	Cell morphogenesis	3.1
1	GO:0031175	Neuron projection development	4.0
1	GO:0007411	Axon guidance	5.2
1	GO:0048666	Neuron development	3.2
1	GO:0048858	Cell projection morphogenesis	3.1
1	GO:0030030	Cell projection organization	2.9
2	GO:0007155	Cell adhesion	2.9
2	GO:0022610	Biological adhesion	2.9
3	IPR002165	Plexin	7.9
3	IPR003659	Plexin/semaphorin/integrin	6.4
3	SM00423	Domain found in Plexins, Semaphorins and Integrins	5.7
3	IPR001627	Semaphorin/CD100 antigen	6.9
3	SM00630	Sema	6.2
4	IPR013032	EGF-like region, conserved site	3.0
4	IPR006210	EGF-like	3.5
4	SM00181	EGF	3.2
4	IPR000742	EGF-like, type 3	3.4
4	IPR002049	EGF-like, laminin	7.7
4	SM00180	Laminin-type epidermal growth factor-like domain	7.0
4	IPR003961	Fibronectin, type III	2.9
5	IPR002909	Cell surface receptor IPT/TIG	8.4
5	SM00429	Ig-like, plexin, transcription factor domain	7.6

Terms beginning with: GO = Gene Ontology term; IPR = interpro; SM = SMART protein domain.

Other genes in the top 25 upregulated genes included *Szt2* and *Sst1*.*1*. *Szt2* mutant mice have a lower seizure threshold [48]. Somatostatin (*Sst1*.*1*) has previously been implicated in decreasing growth as well as decreasing aggressive behavior in fish [49,50]. Social isolation can lead to increased aggression in fish [51]. It would be interesting to determine whether group housed sticklebacks have slower growth and reduced aggression than individually-housed fish. In addition, it would be interesting to manipulate somatostatin levels [49] and determine whether there was an impact on growth and gene expression.

The list of 985 genes upregulated in the hindbrain/cerebellum as a result of group housing included many other interesting genes in addition to those presented in Table 2. We will highlight a few here, although the entire list can be found in S1 File. Many enriched genes were in neurotransmitter or neuromodulator pathways. First, several genes related to acetylcholine synthesis and signaling were higher in group-housed fish: acetylcholinesterase (*Ache*, ENSGACG00000000728; log2FC = 0.9; FDR = 0.009), choline o-acetyltransferase (*Chat*, ENSGACG00000002482; log2FC = 0.8; FDR = 0.008), and the muscarinic acetylcholine receptor, *Chrm2a* (ENSGACG00000019948; log2FC = 1.2; FDR = 0.04) (S1 File). Acetylcholinergic cells are found in cranial sensory and motor nuclei and throughout the reticular formation of the hindbrain [52]. *Chrm2a* also expressed in cranial nuclei [53]. Given these expression patterns, we speculate that increased acetylcholine signaling is related to higher levels of sensory processing due to more sensory stimulation in the group-housing environment.

In addition, galanin receptor (*Galr1*; log2FC = 1.4; FDR = 0.014) and several insulin signaling genes were regulated as a function of social status. Specifically, an insulin receptor (*Insr*, ENSGACG00000010475, log2FC = 1.1; FDR = 0.0003), insulin-like growth factor 2 receptor (*Igf2r*, ENSGACG00000005960; log2FC = 1.1; FDR = 0.016), and two insulin receptor substrate 2 orthologs (*Irs2*: ENSGACG00000014133; log2FC = 0.8; FDR = 0.003; ENSGACG00000003564; log2FC = 0.7; FDR = 0.01) were all significantly higher in the hindbrain/cerebellum of group-housed fish (S1 File). Both galanin and insulin have been implicated in fish feeding [54], so perhaps upregulation of these genes is related to increased competition for food in group housing conditions. In addition, several insulin-related genes are regulated in response to social conditions: *Igf2r* was shown to be increased in brains of subordinate rats [55], and insulin signaling alters social behavior in honeybees [56].

Another signaling pathway gene that was differentially expressed was prostaglandin F2 receptor inhibitor (*Ptgfrn*; ENSGACG00000014419; log2FC = 0.8; FDR = 0.013). Prostaglandin F2α signaling increases fish reproductive physiology [57] and behavior [58]. The females in social groups were exposed to males but isolated females were not, so it may be that mixed-sex housing facilitates reproduction. Investigating levels of reproductive hormones would directly address this question.

Opiate signaling pathway genes were also regulated as a function of social status. Prepronociceptin a (*Pnoca*, ENSGACG00000014805; log2FC = 1.7; FDR = 0.003) and its receptor, opiate receptor-like 1 (*Oprl1*; ENSGACG00000010479; log2FC = 1.1; FDR = 0.02), were both expressed at higher levels in fish in social housing. Interestingly, *Pnoc* and *Oprl1* (aka NOP) were also found to be higher in brains of mice housed in groups than in mice housed in isolation [59]. Nociceptin signaling decreases stress and anxiety in mammals [60]. It may be that social interactions in group-housed fish lead to increased nociceptin signaling, which results in reduced stress and anxiety. Alternatively, individually-housed fish might have decreased levels of nociception signaling.

**Fig 2:**
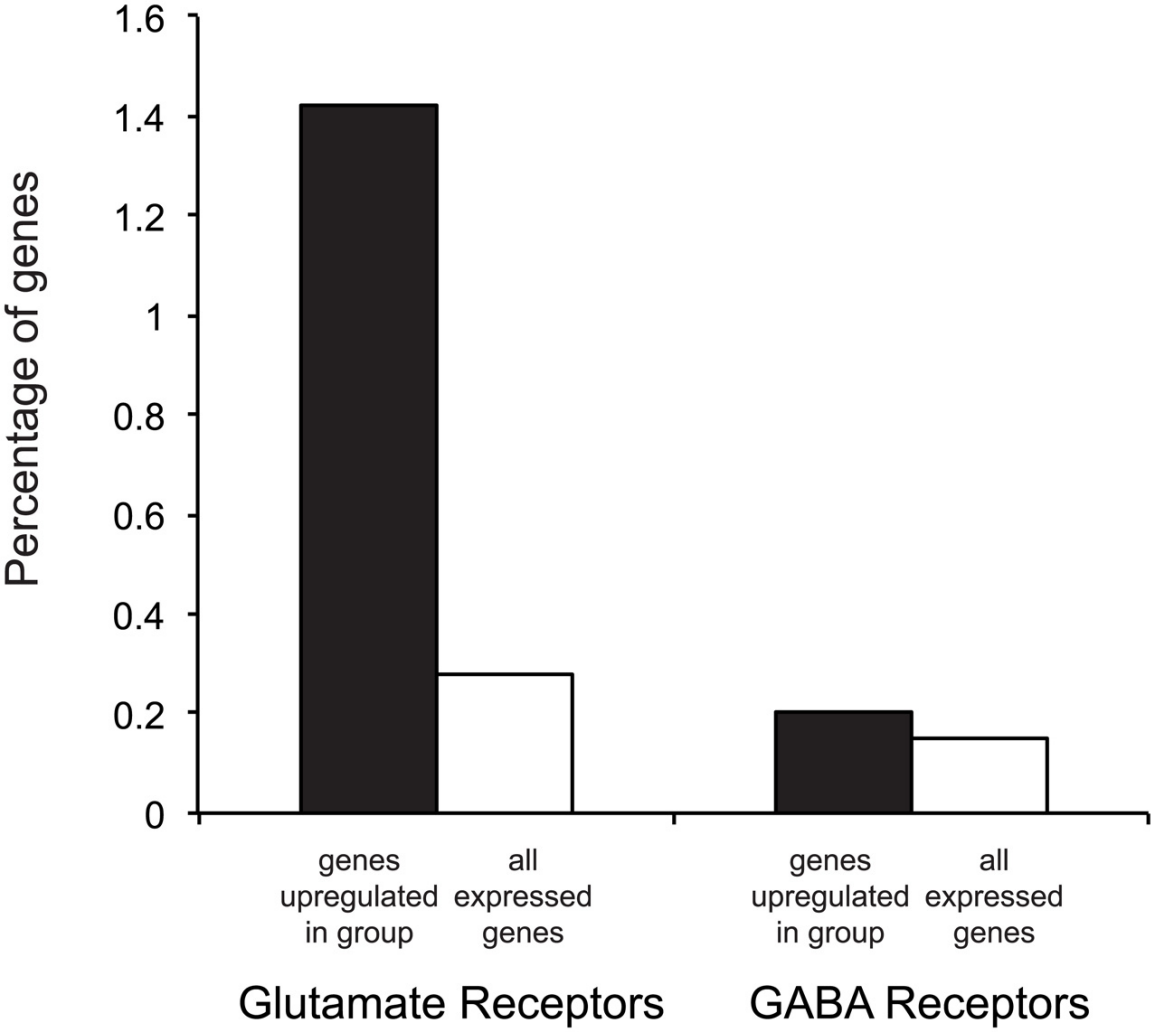
Glutamate receptors are enriched in the list of upregulated genes from group-housed fish. The percentage of genes in the significantly upregulated and total gene list is shown for glutamate and GABA receptors. There is a significant enrichment in glutamate but not GABA receptors in the list of genes upregulated in group-housed fish.

Finally, 14 glutamate receptor subtypes were found in the list of significantly upregulated genes in socially housed fish (S1 File; *Gria1a*, *Gria4b*, *Grik2*, *Grik3*, *Grik5*, *Grin2ab*, *Grin2b*, *Grin2bb*, *Grin2ca*, *Grin2db*, *Grip2b*, *Grm3*, *Grm5*, *Grm8*). Because the glutamate receptor family is quite large, we tested to see whether this was a specific enrichment or was simply a result of there being a large number of glutamate receptor genes in the entire gene list. We also compared the level of enrichment of another large neurotransmitter receptor family, the GABA receptors. This analysis showed that glutamate but not GABA receptors were significantly enriched in fish housed in social groups (Χ^2^ = 43, P < 0.00001; Fig 2).

### Genes upregulated in the hindbrain of individually-housed fish

We next examined genes that were higher in the hindbrain of individually-housed fish (Table 3). The list of the top 25 genes with the highest fold-change contained genes with diverse functions. For example, *Slc16a1* has been implicated in neurogenesis in zebrafish [61]. *Ddit4* may play a role in development through interactions with *Wnt*/*beta catenin* signaling [62]. There were several transcription factors with varied functions (*Tcf24*, *Mxd3*, *Sox1b*). *Gatm* is involved in creatine synthesis. Interestingly, *Mad2l1bp*, which has homology to a gene involved in cell division and the spindle checkpoint pathway, was also found to be regulated by social interactions in other populations of sticklebacks. Specifically, it was higher in males following a territorial intrusion [14]. Finally, novel gene ENSGACG00000001910 has homology to the MHC class 1 antigen family. A gene from this family was previously shown to be expressed at higher levels in brains of female than male cichlids [11].

The entire list of 401 genes upregulated in individually-housed fish included several other genes with interesting functions, and is shown in S1 File. One of these was an enzyme involved in steroid biosynthesis, hydroxysteroid (17-beta) dehydrogenase 7, which was expressed at higher levels (*Hsd17b7*; ENSGACG00000016134; log2FC = 0.6; FDR = 0.02). Hsd17b7 is involved in the biosynthesis of cholesterol and sex steroids, and thus may play a role in regulating steroid hormone abundance in the brain. Another gene upregulated in individually-housed fish, MAD2 mitotic arrest deficient-like 1, was also shown to be higher in brains of isolated rats (*Mad2l1*; ENSGACG00000001594; log2FC = 0.8; FDR = 0.007) [13].

We next performed functional annotation and enrichment analysis of the list of genes upregulated in individually-housed fish. Relatively few categories were enriched, and they included genes related to RNA processing (Table 5).

**Table 5:** Functional annotation and clustering of genes expressed at higher levels in individually-housed fish.

Cluster	Term	Description	Fold Enrichment
1	dre03040	Spliceosome	5.4
1	SM00651	Small nuclear ribonucleoprotein involved in pre-mRNA splicing	33.5
1	IPR006649	Like-Sm ribonucleoprotein, eukaryotic and archaea-type, core	19.5
1	IPR001163	Like-Sm ribonucleoprotein, core	18.0
2	GO:0030529	Ribonucleoprotein complex	3.4

Terms beginning with: GO = Gene Ontology term; IPR = Interpro protein domain; SM = SMART protein domain; dre = KEGG pathway.

## Conclusions

In summary, we found that manipulating social housing impacted the expression of genes predominantly in the hindbrain/cerebellum. In group-housed fish, many of the upregulated genes were in developmental signaling pathways, and functional annotation reinforced the conclusion that there was enrichment of development-related genes in this dataset. These results suggest that fish in group-housing environments experience more neurogenesis or more axon and dendrite outgrowth. Alternatively, because many developmental genes act as repressors, it may be that upregulated expression of these genes is actually associated with decreased neurogenesis. It would be interesting to distinguish between these possibilities by directly by comparing levels of cell division and differentiation on a cellular level. Other differentially expressed genes were involved in stress/anxiety, social behavior, and possibly sensory processing. These findings suggest interesting directions for future research on the molecular control of normal social interactions in sticklebacks and other systems. In the future it could also be interesting to evaluate different timescales of experimental manipulation, for instance social isolation for an entire lifetime or across evolutionary timescales [63].

## Supporting Information

S1 File List of all differentially expressed genes in the hindbrain. File contains a list of all hindbrain genes that were significantly upregulated (FDR < 0.05) in group- and individually-housed fish, on two separate worksheets.
(XLSX)
